# A preliminary study of spinal cord blood flow during PVCR with spinal column shortening: A prospective clinic study in severe rigid scoliokyphosis patients

**DOI:** 10.1097/MD.0000000000021579

**Published:** 2020-08-07

**Authors:** Tao Li, Zhi Zhao, Yingsong Wang, Jingming Xie, Ying Zhang, Ni Bi, Zhiyue Shi, Qiuan Lu, Quan Li

**Affiliations:** Department of Orthopaedic Surgery, Second Affiliated Hospital of Kunming Medical University, Kunming, Yunnan Province, China.

**Keywords:** posterior vertebral column resection, spinal column shortening, spinal cord blood flow, spinal deformity

## Abstract

Posterior vertebral column resection (PVCR) was the most powerful technique for treating severe rigid spinal deformity, but it has been plagued with high neurologic deficits risk. The fluctuations of spinal cord blood flow (SCBF) play an important role in secondary spinal cord injury during deformity correction surgery.

The objective of this study was to first provide the characteristic of SCBF during PVCR with spinal column shortening in severe rigid spinal deformity.

Severe rigid scoliokyphosis patients received PVCR above L1 level were included in this prospective study. Patients with simple kyphosis, intraspinal pathology and any degree of neurologic deficits were excluded. The deformity correction was based on spinal column shortening over the resected gap during PVCR. Laser Doppler flowmetry was used to monitor the SCBF at different surgical stages.

There were 12 severe rigid scoliokyphosis patients in the study. The baseline SCBF was 316 ± 86 perfusion unite (PU), and the SCBF decreased to 228 ± 68 PU after VCR (*P* = .008). The SCBF increased to 296 ± 102 PU after the middle shortening and correction which has a 121% increased comparison to the SCBF after VCR (*P* = .02). The SCBF will slightly decrease to 271 ± 65 PU at final fixation. The postoperative neural physical examination of all patients was negative, and the MEP and SSEP of all patients did not reach the alarm value during surgery.

These results indicate that PVCR is accompanied by a change in SCBF, a proper spinal cord shortening can protect the SCBF and can prevent a secondary spinal cord injury during the surgery.

## 1 Introduction

Severe rigid spinal deformity always indicated more aggressive procedure and potentially higher risks. Posterior vertebral column resection (PVCR) was recognized as the most powerful technique to correct severe rigid spinal deformity, but the incidence of complications of PVCR procedure was not low.^[1–3]^ However, intraoperative neuromonitoring equipment are used for monitor the spinal cord and prevent neurological complications, iatrogenic spinal cord injury (SCI) still occurs during surgeries. Surgeons remain highly concerned the neurological complications of the PVCR procedure when it has been performed by experienced surgical team. In our department, PVCR have been performed to treat severe rigid deformities more than 15 years, the rate of neurological complications was low and no one patients were encountered permanent neurological complication.^[4]^ During PVCR procedure, we emphasized that spinal column shortening can decrease the tension of the spinal cord and increased the compliance of the spinal cord for deformity correction, this could be the reason which can decrease the risk of neurological injury secondary to deformity correction.

Spinal cord blood flow (SCBF) was one of the most important factors in motor and sensor function, it also has a very important role in secondary SCI.^[5–7]^ Laboratory can provide some study about the SCBF in traumatic SCI animal models, but those studies differ from iatrogenic SCI in human surgery.^[8,9]^ Turner et al^[10]^ first reported the SCBF changes in a patient received three-column osteotomy, then Gallagher et al^[11]^ used laser speckle contrast imaging to visualize intraoperative SCBF at traumatic SCI site of patients. However, we are unaware of a study researched the SCBF in spinal deformity patient received PVCR with spinal column shortening. The aim of this study was to first provide the characteristic of SCBF during PVCR procedure in severe rigid spinal deformity patients, and this study was the first study that investigates the SCBF change in spinal deformity surgery.

## 2 Methods

### 2.1 Patients profile

This study involved the severe rigid spinal deformity in patients who received PVCR from July 1, 2017 to May 31, 2018 in a single institution. The patients with Cobb angle of the major curve more than 90° in the coronal plane and the flexibility of the major curve <20% were included in this study. Patients with simple kyphosis and with any degree of neurologic deficits were excluded. Patients who showed chiari malformation, syringomyelia, tethered cord, diastematomyelia, and other intraspinal pathology in CT or MRI were also excluded. The patients in which the vertebral resection was below L1 level were also excluded from this study. Strictly physical examination including systemic neurologic function was performed to evaluate the motor, sensory, reflex function, and pathological sign of all patients before and after surgery. Preoperatively and postoperatively standing anterior–posterior and lateral radiographs of the spine was performed, Cobb's angle was measured at the coronal and sagittal planes. CT-scan was used to evaluate the spinal deformity before surgery, and the shortening distance of the spinal column was measured at CT scan after surgery. CT-scan and MRI were used to evaluate the intraspinal pathology before surgery. Heart rate, blood pressure, oxygen saturation, and hemoglobin were also monitored at different surgical stages during the surgery. All patients included in the study underwent surgery performed by the same team. This study was approved by the Institutional Review Board (IRB) of the Second Affiliated Hospital of Kunming Medical University, and the informed consent forms were obtained from all patients or their guardians.

### 2.2 Anesthesia and PVCR procedure

Similar general anesthesia was used in all patients. Controlled hypotension was banned from using in all patients in this study, an efficient high-dose of TXA (an intravenous loading dose at 100 mg/kg, followed by a continuous infusion of 10 mg/kg/h) were used to reduce the bleeding during surgery. The PVCR procedure was based on standard operating procedure reported by Xie.^[12]^ The patients were placed in prone position on Jackson spine table and a posterior incision was made. Following dissection of the paravertebral tissues, pedicle screws were inserted in fusion levels. Bilateral transverse processes of the resection vertebra were exposed and removed, then the segmental blood vessels of the resection vertebra were exposed and ligatured. Posterior elements including lamina, spinous process, bilateral superior and inferior articular processes were removed. Then the spinal canal was exposed, intraspinal blood vessels were cauterized with bipolar to reduce bleeding. The pedicle, vertebral body and adjacent intervertebral discs were resected carefully to avoid invading the spinal cord. At the completion of VCR, a gap for further correction was created, compression could reduce its height and shorten the spinal column. Using exchanged-rods technique, in situ rod bending and derotation maneuver with at least four times repeated spinal column shortening to correct the deformity and maintain a low tension of the spinal cord.

### 2.3 Monitoring of SCBF

Laser Doppler flowmetry (PERIMED PeriFlux 5000 mainframe) was used to monitor the SCBF, and the blood flow unit parameter is set to 0.2τ. The system includes laser Doppler probe, perfusion monitor, and software. The laser Doppler probe was placed on the midline of dorsal surface of the spinal cord at the level of the VCR, it was needed to avoid the blood vessel on the surface of the spinal cord. The SCBF was acquired by the probe and monitored by perfusion monitor, then the data was described by the software and showed on the computer. The SCBF were monitored at different surgical stages: after laminectomy, after VCR, first, middle and last spinal column shortening and deformity correction, final fixation. SCBF measurements were acquired and processed by the laser Doppler probe and SCBF monitor, and data depicted with computer-based software.

### 2.4 Intraoperative neurophysiological monitoring

All patients have been conducted combined SSEP and MEP monitoring during PVCR procedure, and additional wake-up tests has been performed after final fixation and the end of the surgeries. The Intraoperative neurophysiological monitoring (IONM) (Cascade, Cadwell Co, USA) was performed under the supervision of an experienced neurophysiologic doctor. Warning criteria were defined for both SSEP and MEP data. Ongoing, trial-to-trial, 50% or greater decrease in amplitude was used for SSEP data, a 75% or greater decrease in amplitude was used for MEP data.

### 2.5 Statistical analysis

The data were statistically analyzed by SPSS software package (SPSS, version 22.0, Inc, Chicago, IL). Quantitative data were calculated as mean ± standard deviation. Independent Student's *t* test was calculated. In our study, *P* < .05 was considered statistically significant.

## 3 Results

### 3.1 Demographic characteristics and surgery outcome

Twelve severe rigid scoliokyphosis patients were included in this prospective study. There were 7 males and 5 females in the study, the mean age was 18.7 ± 3.8 years old (range 14–26 years). The mean preoperative scoliosis of 125° ± 17° was corrected to 41° ± 5° (correction rate: 67.2%), and the mean preoperative kyphosis of 108° ± 19° was corrected to 38° ± 5° (correction rate: 64.7%). All patients received only one vertebral body resection between T8 to T12, and only one pair of segmental arteries were ligated at one level. The mean operation duration was 523 ± 72 min and the mean estimated blood loss was 2653 ± 953 mL. The mean distance of the spinal column was 14.8 ± 1.6 mm and was almost 50.6 ± 8.3% of the resection gap distance (Table 1).

**Table 1:**
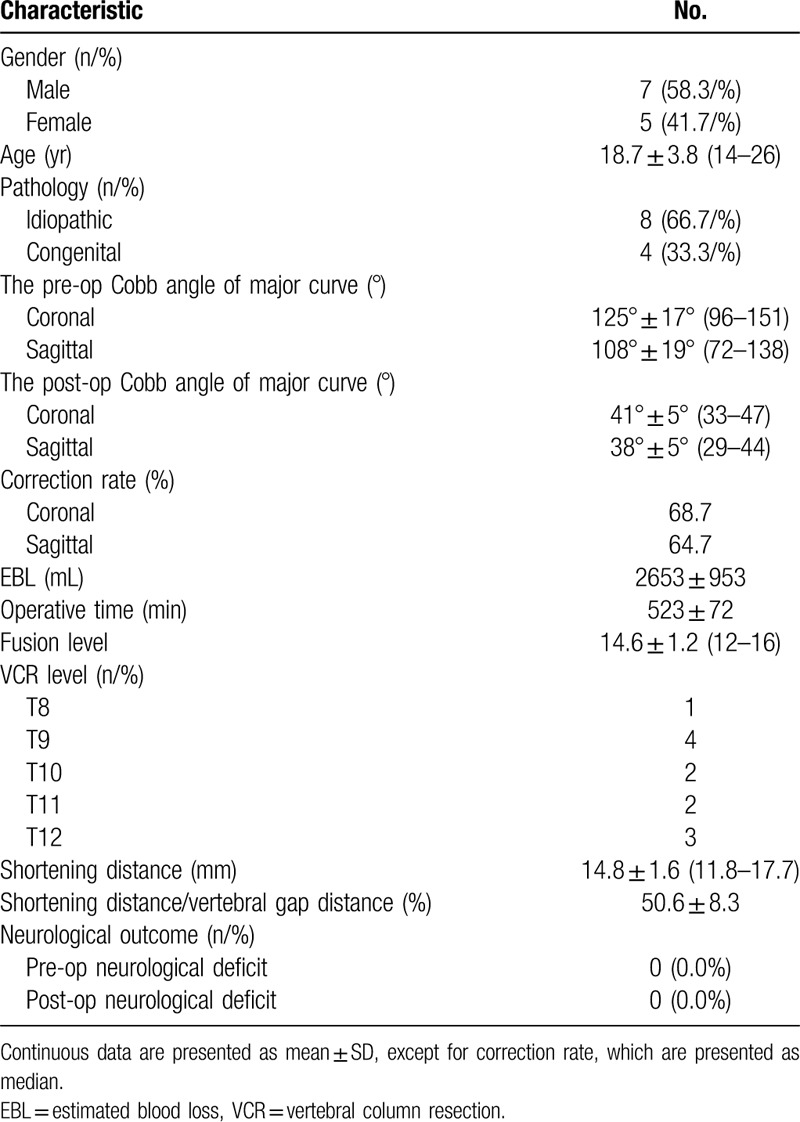
The demographics and surgical trait of the patients.

### 3.2 Measurement of SCBF

**Figure 1:**
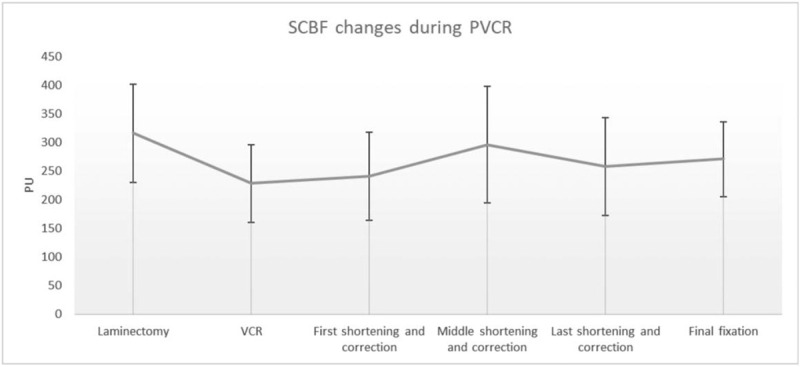
The spinal cord blood flow (SCBF) changes during posterior vertebral column resection (PVCR). The baseline SCBF was 316 ± 86 PU, and the SCBF decreased to 228 ± 68 PU after VCR (*P* = .008). The SCBF increased to 296 ± 102 PU after the middle shortening and correction which has a 121% increased comparison to the SCBF after VCR (*P* = .02). The SCBF will slightly decrease to 271 ± 65 PU at final fixation. PU = perfusion unite.

Baseline SCBF was obtained after laminectomy and before vertebral column resection (VCR), the baseline SCBF was 316 ± 86 perfusion unite (PU); the SCBF decreased to 228 ± 68 PU after VCR, and had a 28% decreased comparison to the baseline SCBF (*P* = .008); The SCBF increased to 241 ± 77 PU after the first shortening and correction, and the SCBF increased to 296 ± 102 PU after the middle shortening and correction which has a 121% increased comparison to the SCBF after VCR (*P* = .02); The SCBF will slightly decrease to 258 ± 85 PU after the last shortening and correction, and then the SCBF maintained at 271 ± 65 PU (Fig. 1). The baseline SCBF between the concave side and convex side of the spinal cord were different, the baseline SCBF on the concave side was higher than convex side in 8 patients (66.6%), but there was no statistically significant difference between SCBF on the concave side and convex side (*P* = .69). There were 9 (75%) patients who presented a higher SCBF on the convex side than the concave after final correction, but there was also no statistically significant difference between SCBF on the concave side and convex side (*P* = .17).

### 3.3 IONM and neurological function

The latencies and amplitudes of SSEP showed no significant change before and after laminectomy, the MEP also showed no significant change. The signal of IONM fluctuates up during VCR and correction period, but the MEP and SSEP all did not reach the alarm value. The wake-up test was performed after final fixation and operating finished by discontinuing the anesthetic agents and all patients could move the toe on command. After surgery, neural physical examination was performed in all patients and none neurological deficit was found.

## 4 Discussion

The treatment of severe rigid spinal deformity has been a daunting challenge for orthopedic surgeon, and extended efforts have been devoted to find a more effective treatment. Suk et al^[13]^ first promoted a posterior-only VCR (PVCR) for severe spinal deformity in 2002. Then, applying PVCR for treating severe and rigid spinal deformity has been used worldwide and gradually recognized by more surgeon.^[1,12]^ Simultaneously, the obvious neurological deficit risk accompanied with the aggressive corrective technique has been emphasized repeatedly.^[3,14]^ Kim et al^[2]^ reported a series of 152 patients undergone PVCR, there were 27 patients with postoperative transient neurologic deficit and 6 patients with permanent neurologic deficit. However, based on our clinical practice and experience, PVCR could not only result in satisfactory correction of deformity but could also decrease the risk of SCI secondary to surgical intervention by shortening the spine column.^[12]^ This was because the appropriately shortening of the spinal column could decrease the tension of the spinal cord and could increase the tolerance of spinal cord for the displacement of the spinal column during deformity correction. Meanwhile, SCBF was one of the most important factors in motor and sensor function, several articles published on the pathophysiology of the spinal cord distraction injury suggest that the underlying mechanism of injury is a microvascular ischemic event. We infer that the spinal column shortening can also protect the SCBF to avoid SCI in PVCR procedure, so we carried on this study to clarify the change of SCBF during PVCR with spinal column shortening and prove our inference.

Previously, the hydrogen clearance method, radioactive tracer microsphere technique, the C14-iodo-antipyrine autoradiography and laser Doppler flowmetry technique have generally been used to measure the SCBF. Some of these measurements, including C14-iodo-antipyrine autoradiography, radioactive tracer microsphere technique and hydrogen clearance technique, cannot be used on humans. Review of the literature, the real time assessments of SCBF used for human are usually performed by laser Doppler flowmetry.^[10,11]^ The laser Doppler flowmetry is a non-invasive, real time, continuous online and convenient technique that can monitor microvascular SCBF.^[15]^ The Laser Doppler flowmetry was a measurement that monitor the blood perfusion of the organic/tissue (microcirculation) not the blood flow of the vessels. So, it is needed to avoid the blood vessel on the surface of the spinal cord. In this study, we adapt this monitoring technique to surgical patients with little risk and can get quantifiable and real time data for comparative analysis.

Surgeon's understanding of the harmful of the distraction to the spinal cord began in 1975, 74 spinal cord injuries due to spinal distraction instrumentation for spinal deformity were reported by SRS.^[16]^ Other author also reported SCI due to distraction instrumentation.^[17]^ Then, surgeon generally realized the threat of distraction to spinal cord in spinal deformity correction and began to focus on the spinal column shortening. Spine column shortening was not a new topic, the technique was sometimes used in the surgery for spinal tumor or other diseases.^[18]^ The core technique in our institution for PVCR is a proper spinal column shortening which can maintain a proper low tension of the spinal cord. In this study, we observed that the SCBF did not drop during deformity correction, indeed it rose with spinal column shortening. This illustrated that the proper low tension of the spinal cord has a protective effect on the SCBF and can maintain the SCBF at a satisfactory level during the deformity correction. It is should be stressed that spinal column shortening was the way that can be used for the correction of a severe rigid deformity without the risk of neurological complications. For non-deformity spinal column, Kawahara and other researchers also indicated that a proper spinal column shortening could not injure the spinal cord and could increase the SCBF in animal experiment.^[19,20]^ In this study, the SCBF could maintain at a satisfaction level to avoid SCI and proved its protection effect of blood flow, although the SCBF did not continued to increase during repeated shortening. The reason is that the correction operation might be harmful to the SCBF and it partially counteracted the benefit of the shortening.

The distance of the spinal column shortening is also a key point, a proper distance can increase the SCBF without neurologic dysfunction. There was no study about spinal column shortening in human body before this study, but some in animal experiment. In this study, the SSEP and MEP of all patients did not reach the alarm value and patients did not showed any neurological deficit with a mean of 50.6% shortening distance. The SCBF also could be maintained at a satisfactory level in this study. So, almost 50% shortening of resection gap distance is benefit for the patients who received one vertebral resection. But it does not mean the more shortening the better, it is not a linear relationship between SCBF and neurologic function. In a canine model study, the SCBF had a maximal increase at 10 mm of shortening, and the spinal cord-evoked potential began to change when the spinal cord had a 15 mm of shortening.^[19]^ In another animal study, shortening within 2/3 of vertebral segment height can increase SCBF, but one vertebral segment height of shortening can decrease SCBF. The author also found that more than 2/3 vertebral segment height shortening can cause an irreversible SCI.^[20]^

Intraopertative bleeding can be massive in severe rigid spinal deformity patients received PVCR. The ligation of segmental arteries and intraspinal blood vessels cautery can reduce the bleeding in PVCR, but it also can influence on the spinal cord blood perfusion. This was one of the important reasons that there was a slightly decrease of SCBF after VCR in this study. Nambu et al^[21]^ has reported that the blood perfusion decreased to 70.13% of the control value after ligation of one pair segmental arteries in dogs, but the spinal cord evoked potentials had no significant changes. Other animal studies also showed that ligation of <5 pairs of segmental arteries, <4 pairs if the Adamkiewicz artery included, would not cause spinal cord ischemia and neurological dysfunction.^[22]^ Combing the result of the IONM in this study, one pair of segmental arteries ligation and one segment of intraspinal blood vessels cautery in patients received PVCR were safe, and has mild harm for the SCBF.

There was a negative correlation between spinal cord tension and SCBF. Based on the results of an in vitro study, Bassi et al indicated that the cord interstitial pressure would increase with the increase of the distraction weight.^[23]^ Peter et al found that the SCBF decrease associated with spinal distraction.^[24]^ Spinal distraction can lead to a high spinal cord tissue pressures and a secondary spinal cord compartment syndrome which have a strong impact on the SCBF. Instead, Phang et al indicated that releasing the spinal dural compression can improve the intraspinal pressure and spinal cord perfusion pressure at the injury site.^[25]^ So, we can summarize a model in PVCR procedure that propel spinal column shortening can decrease the tension of the spinal cord which can decrease the cord interstitial pressure and intraspinal pressure, the low spinal cord tissue pressure can increase or protect the SCBF to avoid secondary SCI. We also think that the PVCR combine with spinal column shortening have a potentially to treatment traumatic SCI.

This study has some limitation. First, this study included a relatively small number of patients. Second, the features of SCBF cannot be associated with pathology because spinal cord specimens of the patients could not be obtained.

## 5 Conclusions

The PVCR process is accompanied by SCBF changes, a proper spinal cord shortening can protect the SCBF and can prevent a secondary SCI during the surgery. The severe rigid spinal deformity can lead an SCBF unbalanced on the convex and concave side of the spinal cord. In the further study, we will increase the number of cases and include the patients with intraspinal pathology.

## Author contributions

**Conceptualization:** Tao Li, Jingming Xie.

**Data curation:** Tao Li, Zhi Zhao.

**Formal analysis:** Tao Li, Zhi Zhao, Ying Zhang, Ni Bi, .

**Investigation:** Tao Li, Zhi Zhao, Zhiyue Shi, Qiuan Lu.

**Methodology:** Yingsong Wang, Ying Zhang.

**Project administration:** Yingsong Wang, Jingming Xie.

**Resources:** Ni Bi, Zhiyue Shi.

**Software:** Qiuan Lu, Quan Li.

**Writing – original draft:** Tao Li.

**Writing – review & editing:** Jingming Xie, Yingsong Wang.
